# Metabolic and Bariatric Surgery in Adolescents

**DOI:** 10.1007/s13679-021-00423-3

**Published:** 2021-03-16

**Authors:** Christopher G. Chalklin, Elizabeth G. Ryan Harper, Andrew J. Beamish

**Affiliations:** 1 Cardiff and Vale University Health Board, Cardiff, UK; 2 Department of Gastrosurgical Research, Institute of Clinical Sciences, Gothenburg University, Gothenburg, Sweden; 3 Swansea University Medical School, Swansea University, Swansea, UK; 4 Department of GastroSurgical Research and Education, Sahlgrenska Universitetsjukhuset, Institute of Clinical Sciences, Gothenburg University, Gothenburg, 41431 UK

**Keywords:** Adolescent, Bariatric surgery, Cardiovascular disease, Childhood obesity, Obesity, Type 2 diabetes

## Abstract

**Purpose of Review:**

The prevalence of obesity is increasing in all age groups. Following its success in adults, and with limited success using conservative therapies, metabolic and bariatric surgery (MBS) is increasingly being utilized in adolescents. This review highlights the current evidence and guidelines supporting its use.

**Recent Findings:**

Safety and efficacy mirror results seen in adults. The most recent evidence, as outcomes enter the long term, suggests that comorbidity resolution, including diabetes and hypertension, can even outperform that of adults. Mental health problems persist despite good weight loss. Overall, the positive early weight and comorbidity outcomes are well sustained into the long term.

**Summary:**

There is a growing need to prevent and treat adolescent obesity. Current evidence supports the use of MBS in adolescents. Ongoing and future studies will provide 10-year outcomes and assist in the refinement of multimodal pathways incorporating MBS for the treatment of severe childhood obesity.

## Introduction

Childhood obesity continues to increase globally [1]. The World Health Organization (WHO) estimates that at least 1 in 5 children is overweight, and 41 million children have obesity [1, 2]. Perhaps worse than this, an estimated 8.5% of adolescents in the USA have severe obesity (BMI ≥ 120% of the 95th percentile), representing 4.5 million 12–19-year-olds [1].

Despite significant investment, both preventative and therapeutic strategies to combat childhood obesity are failing to halt the increase in prevalence, let alone reduce it. Adolescent and childhood obesity interventions frequently fail, either to achieve substantial weight loss or to achieve maintenance of any resultant weight loss [3].

When interventions fail, obesity continues from childhood into adulthood [4, 5], taking with it an expanding array of associated physical and mental health problems. These consequences of obesity include increased risk of premature illness, death, and complex psychosocial issues [6–10].

Surgical programs have been widely implemented in class II or above adult obesity (BMI ≥ 35 kg/m^2^) with weight-related comorbidities, or class III obesity (> 30 kg/m^2^) without, with excellent immediate and long-term outcomes, including extended life expectancy, disease prevention, and improved quality of life [11, 12]. The growing prevalence of severe obesity in childhood and adolescence has sparked more frequent consideration of metabolic bariatric surgery (MBS) in the younger patient [13], and several high-quality studies have prospectively evaluated MBS in adolescents.

This review article aims to evaluate the literature on these surgical interventions, including patient selection, current guidance, outcomes, and complications, providing an update on bariatric surgery in adolescents, with particular attention to recent developments.

## Guidance

In order to maximize benefits and minimize complications of bariatric surgery, appropriate patient selection is important [13, 14••]. Guidance relating to adolescent eligibility largely mirrors that of adults, while taking into account ongoing growth and development by allowing for age and sex norms [3, 13, 14••, 15, 16], and also taking into account the importance of having already developed comorbidities at this young age (Table 1) [14••]. While sexual maturation has been included as a selection criterion in prominent studies of adolescent MBS [17••, 18••, 19••], the most recent guidance advises that neither sexual maturation (Tanner stage) nor linear growth should be used in patient selection [14••].

**Table 1:** Eligibility criteria for adolescent metabolic and bariatric surgery (ASMBS, 2018)

Indications for adolescent MBS	
BMI ≥ 35 kg/m^2^ or 120% of the 95th percentile with clinically significant comorbid condition	
BMI ≥ 40 kg/m^2^ or 140% of the 95th percentile	
Contraindications to adolescent MBS	
Medically correctable cause of obesity	
Substance misuse (ongoing or recent history)	
Planned pregnancy (within 12–18) months or current pregnancy	
Inability to adhere to post-operative dietary and medication regimes, including any medical, psychiatric, psychosocial, or cognitive reason for not being able to do so	

Significant comorbidities are obstructive sleep apnea (OSA), type two diabetes (T2D), idiopathic intracranial hypertension (IIH), non-alcoholic steatohepatitis (NASH), Blount’s disease, slipped upper femoral epiphysis (SUFE), gastroesophageal reflux disease (GERD), and hypertension [10]

Eligibility for adolescent bariatric surgery requires a BMI of ≥ 35 kg/m^2^ (or ≥ 120% of the 95th percentile) with a clinically significant comorbidity, or a BMI of ≥ 40 kg/m^2^ (or 140% of the 95th percentile) [14••]. Using the World Health Organization definition, adolescence is defined as ages between 10 and 19 years [20].

Clinically significant comorbidities include obstructive sleep apnea (OSA), type two diabetes (T2D), idiopathic intracranial hypertension (IIH), non-alcoholic steatohepatitis (NASH), Blount’s disease, slipped upper femoral epiphysis (SUFE), gastroesophageal reflux disease (GERD), and hypertension [14••].

Bariatric surgery in an adolescent requires informed consent from the legal guardian, along with assent from the adolescent themselves. As with adult practice, both the parent and the adolescent should be informed of the risks and benefits of any proposed procedure, alongside the requirements for the post-operative period to ensure good outcomes are achieved. Both the adolescent and the parent/guardian should be assessed for their understanding of these points before proceeding. The involvement of the multidisciplinary team is paramount throughout this process, especially when there is doubt of the parent or caregivers ability to provide informed consent or disagreement between the caregiver and the surgical candidate [14••].

Contraindications to adolescent bariatric surgery also mirror adult guidelines and wider surgical practice. Medically correctable causes of obesity, ongoing substance abuse, and current or planned pregnancy (within 12–18 months) are contraindications, as is any medical, psychiatric, cognitive, or psychosocial condition that would prevent reasonable adherence to any post-operative dietary or medication regimes [14••].

While adherence to the above guidance is recommended, special circumstances such as the younger child, or those with syndrome-related obesity, will often need to be considered. In cases of syndrome-related obesity, such as Prader-Willi syndrome (PWS), there has been evidence to show that outcomes from bariatric surgery are inferior when compared to age- and sex-matched individuals with non-syndrome-related obesity [14••, 21]. There are similar findings from studies looking into hypothalamic obesity [22]. Overall, the evidence base for bariatric surgery in syndromic obesity is thin, with current knowledge based on small sample sizes and far from conclusive [14••, 21]. There are complex ethical issues involved, which relate not only to non-maleficence, or “doing no harm,” but also to autonomy and delivering justice, which it is entirely possible may represent ensuring the provision of surgical treatment in some cases [23]. However, in order to determine the ethical justifications of providing or withholding surgery, it is crucial to scientifically evaluate this area more robustly [24]. These patients should therefore be approached on a case-by-case basis within the multidisciplinary team (MDT), ensuring that benefit outweighs risk whenever a surgical intervention is planned [13, 14••]. Regardless of whether a young person is being considered for MBS, where suspicion of syndrome-related childhood obesity exists, long-standing guidance recommends investigation, including relevant genetic testing, to determine the etiology of their obesity [25].

## Procedures

Three surgical procedures are most commonly performed in adult and adolescent bariatric surgery. These are the Roux-en-Y gastric bypass (RYGB), sleeve gastrectomy (SG), and adjustable gastric band (AGB). RYGB has historically been the most performed procedure in adolescents, but the use of SG has now increased to the point of overtaking as the primary procedure in this age group [26, 27].

RYGB involves reconstruction of the upper gastrointestinal tract in the abdomen. This leads to diversion of ingested nutrients to bypass most of the stomach, all of the duodenum, and the first part of the jejunum. The proximal stomach is divided, leaving a small remnant “pouch,” and a gastrojejunal anastomosis is performed 2 to 3 ft (60–100 cm) along the jejunum. The proximal (biliopancreatic) limb of the jejunum is then divided and anastomosed distally, a further 2 to 4 ft (70–150 cm) along this alimentary limb of the jejunum to form a common channel, where undiluted digestive juices mix with ingested food. The mesenteric defects are generally closed to reduce the risk of internal hernia. Procedural technique is identical in both adults and adolescents although some centers may opt to have a specialist pediatric surgeon present [28].

SG involves removal of a large part of the stomach on its greater curvature side, using a linear cutting stapler. The stomach that remains can only accommodate approximately one-quarter of its original volume.

AGB is the least invasive and now least commonly performed surgical procedure in adults. Some units continue to advocate its use in adolescents on grounds of reversibility, although scarring and possibly some vagal effects may continue after removal. A synthetic band is placed around the proximal stomach, whose diameter can be reduced or increased by inflating and deflating, respectively, with saline via a port sited beneath the skin and subcutaneous tissue on the rectus sheath. The restrictive effect of the band limits the volume that the patient can ingest.

High-quality data to influence procedure choice in adolescents are currently sparse. Well-designed trials are needed to compare the outcomes of these procedures in adolescents [29]. The Teen Bypass Equipoise Sleeve Trial (TeenBEST) randomized controlled trial (RCT) is expected to begin recruitment soon, comparing RYGB and SG outcomes in 14–18-year-olds with a sample size of 116 patients/arm [30]. Outcomes from this trial are expected to contribute to the ongoing development of best practice guidelines within the adolescent age group.

Prior to any procedure, adherence to a strict low calorie diet is recommended for at least 2 weeks. Adult literature has demonstrated adherence to this diet to be associated with reduced complications post-operatively along with reduced perceived surgical technical difficulty [13].

## Mechanisms

Bariatric procedures were originally believed to achieve weight loss by two principal mechanisms. Firstly, limiting the volume of food that could be ingested (restriction); secondly, reducing the absorption of ingested calories (malabsorption). However, it has later become apparent that these are not the dominant mechanisms. There does, however, remain controversy on the exact mechanisms by which MBS work.

All three commonly performed procedures lead to reduced dietary intake and induction of early satiety. However, RYGB and SG cause rapid and marked changes in gut-brain neuronal and hormonal signaling mechanisms, which serve as predominating mechanisms [31, 32]. Both RYGB and SG result in elevated post-prandial circulating levels of satiety hormones peptide-YY (PYY) and glucagon-like peptide-1 (GLP-1) [31–33], which are secreted by distal small bowel L cells [31]. SG also leads to decreased levels of grehlin, a hunger hormone produced in the fundus of the stomach [31].

PYY primarily acts on the hypothalamus and vagal afferent nerves to slow gastric emptying and prolong satiety [34]. Higher circulating levels of PYY have been linked with increased energy usage and weight loss [33]. GLP-1 acts on receptors in numerous areas of the brain, in particular the hypothalamus and brainstem. GLP-1 increases pancreatic insulin production, inhibits release of glucagon, and also slows gastric emptying to maintain satiety [35]. There is evidence from mouse models supporting a role for changes in bile acid levels and the subsequent effect on the gut microbiome in weight loss following MBS [36].

## Setting

In order for surgical interventions to be safe and effective, a suitably experienced multidisciplinary team and appropriate setting are essential [34]. As such, patients who are being considered for surgical intervention require tertiary care, and should be referred to a specialist MBS center [14••]. The staff caring for these patients should include health professionals with adequate experience and expertise in looking after young people with obesity, as well as experience in the pre-, peri-, and post-operative care of MBS patients [10]. ASMBS guidance recommends that the team comprises at least a pediatric or adolescent-trained physician; a psychologist, psychiatrist, or similar behavioral specialist; a moderate or high volume MBS surgeon, either adult or pediatric; and a transition plan into an adult program. A program coordinator is also recommended to handle associated processes, including insurance approval requirements where relevant [14••].

Prior to surgery, patients may need assessment of metabolic, endocrine and lung function, sleep apnea testing, helicobacter pylori testing, and treatment, alongside facilities to measure body composition, bone density, and indirect calorimetry [13], although which individuals absolutely require such investigations is not yet clear. Centers offering MBS should have access to these facilities, and must be able to offer frequent follow-up in the early post-operative period, including emergency care provision with access to MBS expertise [13].

## Weight Outcomes

In adults, it is widely recognized that bariatric procedures yield excellent results in terms of weight loss and resolution of comorbidity. A meta-analysis by Chang et al. [37] in 2014 showed that, across 164 studies examining a total of 160,000 patients, BMI 1 year post-bariatric surgery decreased by at least 11.8 kg/m^2^, with good weight loss maintenance to 5 years.

**Fig. 1:**
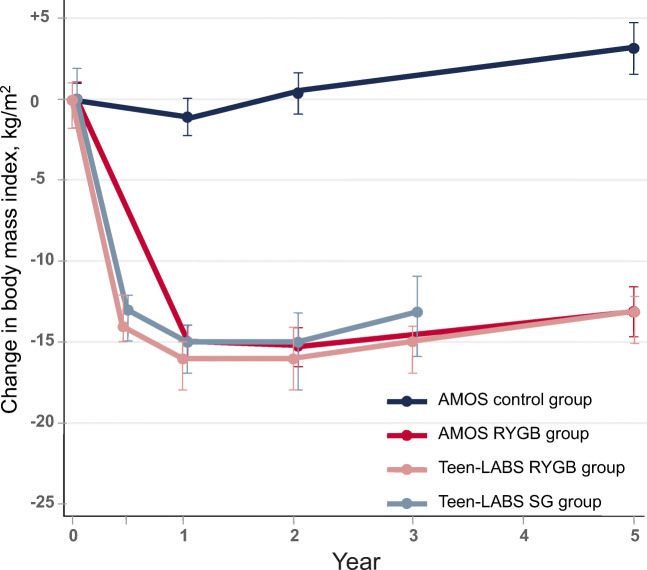
Change in BMI in the Teen Longitudinal Assessment of Bariatric Surgery (Teen-LABS) study and Adolescent Morbid Obesity Surgery (AMOS) study

With increasing utilization of bariatric procedures in adolescents comes growing literature reporting patient outcomes. Similar body weight and BMI reductions to those seen in adults are being demonstrated [13, 37, 38]. As with adult outcomes, BMI reduction varies by procedure, with weight loss at 6 months averaging 11.6 kg/m^2^ following AGB, 14.1 kg/m^2^ following SG, and 16.6 kg/m^2^ following RYGB [13]. Long-term data show that this can be maintained at 5 (13.1 kg/m^2^) and 8 years (17.0 kg/m^2^) following RYGB [16, 17••, 39]. BMI outcomes from two high-quality prospective studies of adolescent bariatric surgery are illustrated in Fig. 1.

## Cardiometabolic Outcomes

While weight loss and BMI outcomes are important indicators of success in adolescent bariatric surgery, the intention to prevent or reverse disease processes is also of great importance. Individuals eligible for MBS often already have comorbid diseases such as dyslipidemia, T2D or glycemic dysregulation, fatty liver disease, and hypertension, alongside depression, anxiety, and psychosocial issues [13, 40]. Cardiometabolic outcomes and cardiovascular risk factors are, therefore, key areas for outcome reporting, centrally embedded in the adult core outcome set for MBS [41] and widely studied within adolescent MBS research.

There is strong epidemiological evidence that children with overweight often retain obesity into adulthood [4]. Building on findings from the end of the twentieth century [34], recent data have emerged in a study by Twig and colleagues [9], who examined 2.3 million adolescents in Israel over a 43-year period (> 42 million person-years). This study found a 3.5 times greater risk of cardiovascular death in individuals who had obesity in late adolescence (BMI ≥ 95th percentile), compared with normal weight (BMI 5th–24th percentiles). Among these individuals with adolescent obesity, the mortality risk from coronary heart disease was almost 5 times that of individuals with normal weight [9]. The group also demonstrated an 8-fold increase in risk of mortality from T2D in those with obesity in adolescence [10]. These findings further highlight the importance of identifying and implementing early intervention strategies in obesity.

Adolescent bariatric surgery has been highly successful in reducing cardiometabolic risk factors and disease, as evidenced across a number of ongoing and completed studies. The Teen Longitudinal Assessment of Bariatric Surgery (Teen-LABS; NCT00565829) is an ongoing prospective multicenter longitudinal study following 242 adolescents undergoing MBS (RYGB, SG, or AGB) for severe obesity. The baseline parameters for this cohort demonstrated a high prevalence of cardiometabolic risk factors and disease [26]. These included hyperinsulinemia (74%), impaired fasting glucose (26%), T2D (14%), elevated high-sensitivity C-reactive protein (hs-CRP) (75%), dyslipidemia (50%), elevated blood pressure (hypertension) (43%), and insulin resistance (71%) [39]. Longitudinal analysis of this study group has shown that the greater the post-surgical weight loss, the more likely the reversal of risk factors and disease processes (dyslipidemia, hypertension, hyperinsulinemia, and T2D). This was sustained at 3 and 5 years following surgery [19••, 39], and Teen-LABS will be examining these outcomes at 10 years.

The Adolescent Morbid Obesity Surgery study (AMOS; NCT00289705), based in Sweden, is a prospective non-randomized comparative observational study. AMOS examines the long-term safety and efficacy of RYGB in 80 adolescents with severe obesity, compared with a matched cohort of non-surgically managed adolescent patients with severe obesity, and an adult cohort also undergoing MBS for severe obesity. Cardiometabolic disease and risk factors were again present at baseline, mirroring the Teen-LABS study: hyperinsulinemia (71%), impaired fasting glucose (20%), T2D (4%), elevated hs-CRP (87%), dyslipidemia (69%), elevated blood pressure (15%), and elevated liver enzymes (31%) [16]. Rates of resolution of these risk factors at 5 years further emphasize the benefit of offering MBS within this age group: 100% resolution of T2DM, impaired fasting glucose and hypertension, 94% resolution of fasting hyperinsulinemia, 92% resolution of impaired liver enzymes, 83% resolution of dyslipidemia, and 74% resolution of elevated hs-CRP. A ten-year data from the AMOS study will be forthcoming regarding long-term outcomes in cardiometabolic risk factors in the post-MBS cohort [40, 43].

The Follow-up of Adolescent Bariatric Surgery study (FABS 5+; NCT00776776), a completed US study, used longitudinal observation methodology to assess long-term safety and efficacy in 58 patients below 21 years of age who had undergone RYGB. The patients selected all had severe obesity, with a baseline BMI of greater than 40 kg/m^2^. Baseline prevalence of cardiometabolic risk factors was similar to those of Teen-LABS and AMOS: 86% of patients had dyslipidemia, 47% had hypertension, and 16% had T2D. Patients were followed to between 5 and 12 years after RYGB, with good resolution rates at both checkpoints: T2D resolved in 88% of patients, hypertension in 76% of patients, and dyslipidemia in 64% [18••].

The principal findings from these three high-quality studies are summarized in Table 2.

**Table 2:** Summary of cardiometabolic outcomes following adolescent metabolic and bariatric surgery in the AMOS, FABS 5+, and Teen-LABS studies

Variable	Baseline	2–3 years	≥ 5 years	Resolution at maximal follow-up
*n*	58–242	81–242	58–139	–
Sex (f)	64–78%	65%	64–79%	–
Age (mean, years)	16.5–17.1	18.5–20.0	21.9–25.1	–
BMI (mean, kg/m^2^)	46–59	30–38	32–42	–
BMI reduction (mean, kg/m^2^)	–	15	13–17	–
Hyperinsulinemia	71–74%	0–21%	4%	79–94%
Impaired fasting glucose	20–26%	2%	0%	76–100%
Type 2 diabetes	4–16%	0%	2–100%	86–100%
Elevated hs-CRP	59–87%	11–25%	25%	71–74%
Dyslipidemia	36–86%	29%	6–38%	64–83%
Elevated blood pressure	15–57%	8%	3–16%	68–100%
Elevated liver enzymes	31%	–	–	92%
Abnormal kidney function	17%	1%	–	86%

Definitions as described in individual studies

*AMOS* Adolescent Morbid Obesity Surgery study; *FABS 5+* Follow-up of Adolescent Bariatric Surgery study; *Teen-LABS* Teen Longitudinal Assessment of Bariatric Surgery study; *BMI* body mass index; *hs-CRP* high-sensitivity C-reactive protein

## Complications and Long-Term Outcomes

As with any medical or surgical therapy, there are inherent risks associated with MBS. The advent of laparoscopic surgery has seen the 30-day mortality rate of MBS fall to around 0.2% in adults [44]. To date, there is only one reported death within 30 days, in an adolescent patient undergoing MBS [45]. Rates of early complications, both major and minor, are low at 0.5% and 0.8% respectively [26]. There are several recognized post-operative complications that may require further operative procedures. The rate of reoperation in the 5 years after RYGB appears to be slightly higher in adolescents than that in adults (20–25%) [45, 46], which may be in part related to the close scrutiny of these young people in research settings, alongside a potentially lower threshold for intervention in the young. Since the initiation of the high-quality studies of adolescent MBS, changes in operative technique and post-operative management have led to substantial reductions in the major causes of reoperation after MBS [47]. Around half of reoperations were for symptomatic gallstone disease, and administration of ursodeoxycholic acid for 6 months has been shown to lead to an 80% reduction in cholecystectomy rate following MBS, although this is not standard practice [48]. In addition, closure of mesenteric defects has led to a 50% reduction in small bowel obstruction requiring reoperation after RYGB [49]. It is likely, therefore, that rates of reoperation will decrease from those in the existing adolescent literature.

In their FABS 5+ study, Inge and colleagues [39] found a mean BMI reduction of 29.2% and significant improvements in associated metabolic comorbidities. Two patients died during follow-up, one due to infectious colitis, at 9 months post-operatively, and the second from substance abuse, 6 years after their operation. Around two-thirds of patients were found to have low iron and ferritin levels and 46% had clinical anemia. Low vitamin D levels were reported in 78%. These findings were closely mirrored by findings in the AMOS study, wherein 61% had iron deficiency and 80% had vitamin D insufficiency at 5 years. Notably, control participants in AMOS also had excess rates of iron deficiency (12% in females) and vitamin D insufficiency (57%) [50]. Other deficiencies have been demonstrated in vitamins A, B1, B6, and B_12_ and folate when patients do not adhere to prescribed supplements [39].

Adolescents have been shown to experience substantial decreases in bone mineral density (BMD) across 2 [51] and 5 years (unpublished data) after RYGB, with the sharpest decrease occurring in the first year and a marked attenuation of decline in subsequent years. These decreases are generally from abnormally high BMD levels to the normal for age and sex, although a subset of around 10% seems to reach an abnormally low BMD. This is an area warranting further investigation in the context of mounting long-term evidence of an excess fracture risk in adults who have undergone RYGB [52].

The Teen-LABS group recently assessed 5-year gastrointestinal symptoms following adolescent RYGB or SG [53]. They found that both procedures were associated with increased rates of nausea, bloating, and diarrhea. However, SG participants had a markedly greater risk of gastroesophageal reflux symptoms (GERS) (relative risk 4.85). The authors concluded that patients needed to be appropriately counseled pre-operatively and monitored post-operatively for GERS.

In their recent 5-year follow-up paper, the Teen-LABS study’s outcomes following RYGB in adolescents (aged 14–18 years) were compared with those of adults (aged 25–50 years) [19••]. This report demonstrated similar weight loss (26% vs. 29%), and highlighted more favorable T2D and blood pressure outcomes in adolescents, illustrated by a significantly higher rate of remission of T2D (86% vs. 53%) and hypertension (68% vs. 41%) in the adolescent group at 5 years. This is particularly important in the knowledge that youth-onset T2D is a far more aggressive subtype than that seen to occur during adulthood [54, 55]. The Teen-LABS group and other commentators have discussed mechanisms to explain this effect, including the potential for early intervention to salvage pancreatic islet function preventing irreversible beta cell injury, and to prevent vascular remodeling and consequent arterial stiffness [42, 54, 56]. All-cause 5-year mortality was similar between groups, but Inge and colleagues [39] have appropriately drawn attention to two adolescent deaths that resulted from substance misuse, which is known to increase following MBS in adults [57, 58], but is not well understood in adolescents, some of whom are alcohol and other substance naïve prior to MBS, others of whom have a past history of substance abuse [59•].

Mental health represents an extremely important domain in this psychosocially vulnerable young population and has recently gained substantial attention with the publication of 5-year mental health outcomes from the AMOS group [59•]. The proportion of patients prescribed psychiatric drugs was similar between the group undergoing RYGB and the control group across 5 years, as were the inpatient psychiatric care requirements in each group, which notably included 2 attempted suicides. Järvholm and colleagues [59•] showed that patients who had undergone RYGB experienced a significant improvement in their self-esteem, anxiety, depression, anger, and binge eating. Crucially, however, overall mood score was no better at 5 years than baseline, and the authors concluded that mental health problems persist, despite significant weight loss.

An unfortunate consequence of major weight loss by any mechanism is excess skin. This is often troublesome, causing a burden not only of psychological distress but also being prone to pain, infection, and ulceration [60]. A study involving AMOS participants compared the burden between adolescents and adults following MBS. Contrary to expectations, adolescents’ skin did not retain elasticity better than adults and return more readily to the new body shape. Instead, adolescents and adults experienced similar problems and desired body-contouring surgery in similar numbers [60]. Relatively few adolescents who underwent bariatric surgery actually accessed subsequent body-contouring surgery (13%), and this was often for pannus-related skin conditions rather than psychological reasons [61].

## Conclusions

The prevalence of obesity in childhood and adolescence is increasing globally and interventions, both for prevention and treatment, have had limited success. Severe obesity presents a substantial burden of concomitant and future consequent disease for individuals and health care systems.

Rapid advances in safety and efficacy in MBS in adults have led to increasing utilization of MBS in the adolescent age group with comparable results, both desirable and undesirable. Evidence in the literature to date demonstrates a good safety and efficacy profile in adolescent MBS and is beginning to support intervening early to prevent the development of irreversible end organ injury.

Ongoing and emerging high-quality studies promise exciting future data, particularly regarding long-term outcomes, with documented commitment to explore in detail the detrimental effects in order to improve the patient experience from this type of intervention. Additional future work should also explore the combination of lifestyle, medical, surgical, and other therapies in the same way oncological management pathways have developed in recent decades, optimizing the role of surgery in the multidisciplinary management of this complex and debilitating disease.
